# Scaling up an Empirically Supported Intervention with Long-Term Outcomes: the Nationwide Implementation of GenerationPMTO in Norway

**DOI:** 10.1007/s11121-019-01047-9

**Published:** 2019-08-23

**Authors:** Elisabeth Askeland, Marion S. Forgatch, Anett Apeland, Marit Reer, Anette A. Grønlie

**Affiliations:** 1 The Norwegian Center for Child Behavioral Development, NUBU, Postbox 7053 Majorstuen, 0306 Oslo, Norway; 2 Implementation Sciences International, Inc. & Oregon Social Learning Center, Eugene, OR 97401 USA

**Keywords:** Adoption, Sustainability, Reach, Fidelity, Implementation, GenerationPMTO

## Abstract

Effective implementation outcomes are necessary preconditions for effective service and positive treatment outcomes for children with behavioral problems. The aim of this study is to assess outcomes of the transfer of the empirically supported intervention GenerationPMTO from the developer in the USA to a nationwide implementation in Norway. Adoption, sustainability, reach, and fidelity are tested across seven generations of therapists in Norway. Participants in the study were 521 therapists who began training in the program. The developer’s team trained the first generation (G1) and the Norwegian team trained the next six generations (G2–G7). The mean rate of certification was 94.2% (*n* = 491). Intervention fidelity was assessed from 1964 video recordings of intervention sessions submitted for certification evaluation using the observation-based measure Fidelity of Implementation Rating System (FIMP). A small but significant drop in fidelity scores was previously observed from G1 to G2; however, fidelity scores recovered at or above G1 scores for G3 Forgatch and DeGarmo (*Prevention Science 12*, 235-246, 2011). Twenty years since the inception of implementation, 314 certified therapists practice the model today, a retention rate of 64%. The outcomes show sustained fidelity scores across seven generations, increasing heterogeneity among therapists trained, and a shift of focus in the target population from clinical to primary services. The present study contributes to the field with the systematic evaluation of outcomes for the full transfer implementation approach with continuing adoption and sustainability, increasing reach and sustained intervention fidelity across several generations of practitioners.

The era of developing and testing parent training programs that address children’s behavior problems has yielded a rich crop of empirically supported interventions (ESI; see Weisz and Kazdin 2017). Programs meeting ESI criteria include Family Check Up (Dishion et al. 2014); GenerationPMTO, which rebranded in 2017 from Parent Management Training - Oregon Model/PMTO® (Forgatch and Patterson 2010); Incredible Years (Webster-Stratton et al. 2001); Parent-Child Interaction Therapy (Eyberg et al. 2001); and Triple P (Sanders and Murphy-Brennan 2010). The combined outcomes from these carefully evaluated programs show that strengthening parenting practices produces multiple positive child outcomes, including reductions in externalizing and internalizing behavior, delinquency, police arrests, deviant peer association, drug use, and out-of-home placement, as well as improved social skills and academic functioning. In the twenty-first century, we must install ESIs within community service agencies using strategies that ensure extended reach with sustained intervention fidelity and positive outcomes for families (Forgatch et al. 2013; Weisz et al. 2014). We must also evaluate implementation strategies and outcomes using the same rigorous standards applied in efficacy and effectiveness research (Herschell et al. 2004).

Transfer of an ESI from developer to community practice is a delicate process that can perish in the face of many obstacles (Kazdin 2013). The following questions point to issues that implementation studies must address. Can an intervention created and tested in a research context produce equivalent outcomes when employed in community settings with diverse cultures and populations? Can training programs produce substantial numbers of certified therapists to practice the program, use it with fidelity with their clientele, and maintain quality assurance? Can the services be provided by practitioners with diverse professional backgrounds within a variety of service agencies? Are communities able to expand their reach to serve all families who seek services? Is it possible for an intervention to adapt to local contexts yet retain fidelity to the core components that produce positive outcomes? These questions direct attention to the need for adoption at community, agency, practitioner, and client levels. Implementation science follows an iterative process that travels through several stages of development (Forgatch et al. 2013; Saldana et al. 2014). We address some of these questions with findings from a nationwide implementation two decades after initiation.

## Implementation of GenerationPMTO in Norway

In 1999, two Norwegian Ministries, Family and Children Affairs and Social and Health Affairs, collaborated to carry out a nationwide implementation of GenerationPMTO. The strategy involved a combination of “top-down” and “bottom-up” approaches: The governmental initiative and long-term funding led from the top, while local services chose whether to adopt the program, recruit and train therapists, and maintain them as certified practitioners in their agencies. A white paper specified a plan to ensure model competence in child mental health, child welfare, and community services in local municipalities. GenerationPMTO was selected as part of this initiative. A goal of the Ministries was to obtain full transfer of GenerationPMTO from the model developers to the Norwegian Center for Child Behavioral Development (NCCBD), a national center for implementation and research that has been the stable base for the nationwide implementation of GenerationPMTO for nearly two decades. The full transfer approach to implementation grew out of this collaboration.

In full transfer, the program developer trains a first generation (G1) of practitioners to certification and gradually turns over program authority to the adopting community. From the G1 certified GenerationPMTO therapists, leaders were selected to form an infrastructure to carry the program forward to future generations of practitioners, trainers, coaches, and fidelity raters (Forgatch and DeGarmo 2011; Forgatch et al. 2013). The present study evaluates long-term implementation outcomes of the full transfer approach of GenerationPMTO in Norway.

During the beginning stages of the implementation, the team at the NCCBD collaborated with the developer to design the infrastructure and complete several activities. Briefly, the start-up activities included specifying goals, formulating agreements, and managing logistical issues, all carried out in an atmosphere of mutual respect, massive support, and much fun. This early work has been described in a series of articles (Forgatch and Gewirtz 2017; Forgatch and Patterson 2010; Forgatch et al. 2013; Ogden et al. 2005; Patterson 2005).

From the G1 Norwegian therapists trained by the developer’s team, the leader of the child department at NCCBD selected 10 of the most qualified certified therapists to form a group called the National Implementation Team (NIT). The NIT serves as the kingpin for the implementation in Norway, acting as the executive governing authority for PMTO. When the developer rebranded to GenerationPMTO, the Norwegian program retained the original nomenclature, PMTO. NCCBD made a long-term strategic plan to conduct future implementation activities in Norway. These activities include the training, coaching, evaluation of intervention fidelity, monitoring of practice, expansion of subsequent generations of PMTO practitioners, and adaptations for specific contexts and populations.

Findings for the first three generations of therapists trained in this full-transfer approach were reported in Forgatch and DeGarmo (2011). The authors had hypothesized that fidelity scores attained by G1 would decline in subsequent generations; the assumption was that training conducted by the implementation sites would not match that provided by the developer’s team. Indeed, there was a significant decline in fidelity from G1 to G2. However, contrary to the hypothesis, G3 fidelity scores returned to G1 levels, indicating the adopting site’s capacity to sustain intervention fidelity (Forgatch and DeGarmo 2011). In the present paper, we extend these findings with fidelity data for four additional generations with fidelity scores at certification. We hypothesize that levels of fidelity will sustain at or above those attained by G1 and G3.

### Implementation Outcomes

Implementation research examines processes and strategies involved in the installation of evidence-based interventions in usual care settings. Proctor et al. (2011) categorized eight implementation concepts to be empirically tested as outcomes: acceptability, adoption, appropriateness, feasibility, fidelity, implementation cost, penetration (also called reach), and sustainability. They made the point that the relevance of outcome variables changes according to the stage of implementation, and there is some overlap in outcomes. For example, acceptability, appropriateness, and feasibility are conceptually related and may be most relevant during the start-up stages; presumably, programs will not be adopted and sustain if they are a poor fit for the agencies, practitioners, and the families they serve. In the present study, we focus on longer-term outcomes of adoption, sustainability, reach, and fidelity.

### Hypotheses

The aim of the Norwegian implementation has been to install and extend the nationwide reach of the PMTO model to diverse agencies and families seeking help while sustaining intervention fidelity and positive family outcomes. Successful outcomes require recruiting, training, coaching, and sustaining substantial numbers of certified practitioners who practice the program with high fidelity to the intervention. We present data across seven generations of Norwegian practitioners who initiated training in the PMTO model with data from 1964 observations of 491 PMTO therapists across diverse service systems throughout the nation.

We test the following hypotheses:

1. *Adoption:* Total numbers of therapists and participating agencies increase with each generation, and percentages of therapists who complete training with certification are at or above the G1 level.
2. *Sustainability:* Therapists continue to practice PMTO with certification and agencies continue their participation.
3. *Reach:* The shift in focus from clinical to preventive services called for less educational requirements of the practitioners and broadening types of community service agencies. Thus, we hypothesize higher percentages of therapists with less educational level and greater participation of organizations providing primary service in the municipalities.
4. *Fidelity:* Fidelity is sustained at or above G1 levels across seven generations with the exception of the previously reported dip for G2. In the earlier report, we anticipated continuing decline in fidelity following the transfer. Given the recovery for G3, we now hypothesize that fidelity will be sustained through G7.

## Method

### Procedure

#### Full Transfer of Implementation

The transfer of PMTO from the program developer to Norway began as the developer’s team initiated training the G1 clinicians in 1999 (Forgatch and DeGarmo 2011). From this group, the NIT was formed as the executive governing authority for subsequent PMTO activities within Norway.

Based on manuals from previous PMTO efficacy trials, the NIT team manualized the training program in a Norwegian handbook and adapted language and metaphors to fit Norwegian culture (Askeland et al. 2010). The NIT then trained the G2 practitioners, primarily from child mental health outpatient clinics and child welfare services at the national level. After G2, a governmental white paper called for a shift, moving much of the resources and efforts from clinical service systems to strengthen efforts within primary services in the municipal communities. Subsequently, G3 through G7 practitioners were recruited mainly from primary services.

**Fig. 1:**
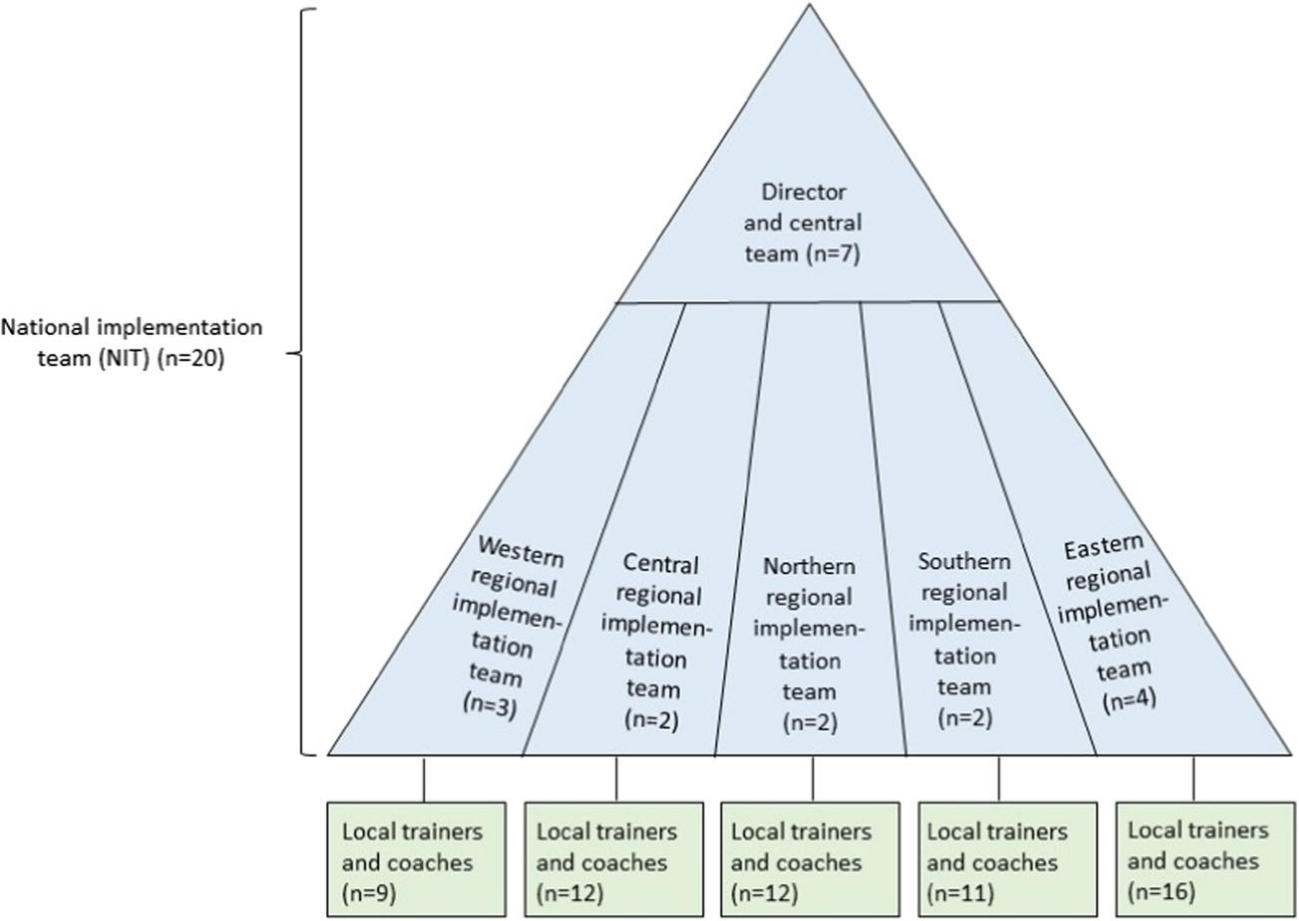
Structure of the National Implementation Team (NIT)

Today, the NIT comprises 20 members, including the Director, a central team, and five regional implementation teams. The regional teams are responsible for their region’s training, coaching, certification, implementation activities, and follow-up of PMTO sites and therapists. In addition, approximately 60 PMTO therapists, led by their regional implementation team, serve as trainers and coaches. See Fig. 1.

#### Brief Description of GenerationPMTO

GenerationPMTO is an evidence-based parent-training program developed to address child and youth behavior problems (Forgatch and Patterson 2010; Forgatch and Domenech Rodríguez 2016; Forgatch and Gewirtz 2017). GenerationPMTO is theoretically based on the Social Interaction Learning model, which specifies that behavior is shaped through reinforcing contingencies provided during interactions with key persons in the social environment (Forgatch and Patterson 2010; Patterson 2005). The parents are regarded as the primary socializing agents for children; thus, the intervention targets parents as the agents of change by teaching parenting skills to prevent and reduce coercive family interaction and replace coercion with positive parenting practices. The core components are positive involvement, skill encouragement, limit setting, monitoring, and problem solving. To facilitate the core components, supporting components are included, such as clear directions, emotion identification and regulation, communication skills, and home-school support (Forgatch and Kjøbli 2016; Forgatch and Patterson 2010; Forgatch and Gewirtz 2017). In addition to core and supporting components, GenerationPMTO emphasizes active teaching and sophisticated clinical process skills to ensure a strong therapeutic alliance with the family (Forgatch and Domenech Rodríguez 2016). In Norway, PMTO practice is limited to families with a focal child between 3 and 12 years old.

#### The Norwegian PMTO Training and Coaching Program

The training program lasts for 18 months and consists of 21 days of workshop training and 1 day of group coaching every fortnight, for a total of 24 days. Coaching is based on video recordings of family sessions. Families are recruited from the ordinary caseload of the trainees, who must work with a minimum of five families who complete the intervention. Training and coaching are based on active teaching skills including role play, problem solving, and other interactive strategies. After certification, therapists join 16 days of in-depth group coaching for 2 years. Following that, they join maintenance coaching groups that meet a minimum of 3 days yearly.

### Participants

Participants were professionals who initiated a PMTO training program between 1999 and 2017. Data are based on these seven generations of trainees (*n* = 521).

#### Educational Background

Educational background incorporates two categories. In Category 1, most therapists had a minimum of a 6-year university education with advanced training in psychology or psychiatry; some had a master’s degree in psychology or social work. In Category 2, most therapists held a 3-year bachelor’s degree in child welfare, social work, teaching, or nursing.

#### Service Systems

In Norway, various systems are divided into different agencies that serve children and youth with or at risk for behavior problems. At the national level, clinical services are divided into child mental health and child welfare. Child mental health is responsible for assessment and treatment of child and youth psychiatric disorders. Child welfare serves at-risk children and families to improve parenting and life skills. At the community level in the municipalities, primary services are divided into three service systems: school counseling, primary health care, and child welfare, all of which mainly provide preventive interventions.

#### Gender and Age

The majority of participants were female, with the percentage increasing from 62 to 100% over generations, reflecting the makeup of gender differences in the primary services. Practitioner age was stable across generations (mean range 43–46).

### Measures

#### Adoption

Adoption has been described as the *decision* to implement a program (Fixsen et al. 2005), an early step in the process. A more encompassing definition relevant for early to mid-implementation stages includes the *uptake* of an intervention (Proctor et al. 2011). This indicates that stakeholders and clinicians employ demanding new programs. We suggest that sustainable implementation includes continuing adoption by practitioners and agencies, which is relevant in later stages of implementation. We assess adoption as uptake in terms of numbers and percentages of practitioners who complete training and numbers of agencies that join the implementation.

#### Sustainability

Proctor et al. (2011) define sustainability as “the extent to which a newly implemented treatment is maintained or institutionalized within a service setting’s ongoing stable operations” (p. 70). Sustainability has also been conceptualized as long-term viability when innovations become integral within organizations (Steckler et al. 1992). We assess sustainability as therapists’ continued PMTO practice in terms of number and percentage of active therapists and number of years of certified practice as well as number and percentage of agencies’ continued participation.

#### Reach

This construct is similar to what Proctor et al. (2011) refer to as “penetration,” defined as the integration of a practice within a service setting and its subsystems, including its spread and service access. We have conceptualized this definition to include diversity in practitioners’ educational backgrounds and the types of agencies serving families. Such diversity can increase access to both primary and clinical services and thereby extend reach. Our definition, therefore, includes increased diversity in therapists’ educational background and increased diversity of service agencies. A relevant aspect within full transfer is that the early generations were expected to lead subsequent generations, which affected selection criteria.

#### Fidelity

Fidelity has been defined as the degree to which an intervention is implemented with adherence and delivery quality as intended by the program developers (Proctor et al. 2011). We assessed PMTO fidelity with the Fidelity of Implementation Rating System (FIMP; Knutson et al. 2009). FIMP evaluates adherence to practices and procedures as described in the manuals and competent application of clinical process and active teaching skills. Scored from video recordings of family or parent-group sessions, FIMP evaluates five categories described here briefly:

1. *Knowledge:* Demonstrates understanding of PMTO content and theoretical principles.
2. *Structure:* Ability to accomplish agenda activities and goals while addressing family issues.
3. *Teaching:* Proficiency in strategies that promote parents’ mastery and use of PMTO practices, balancing verbal teach (give information) and active teach (engage families in the learning process by role-playing, problem solving).
4. *Process:* Promotes a safe and supportive learning context.
5. *Overall Development:* Promotes growth in PMTO use, therapeutic relationship, apparent satisfaction, managing difficult contexts/issues.

The FIMP manual defines each category’s key features, provides rating examples and guidelines, and details scoring procedures. Categories use a nine-point scale, 1–3 indicates “needs work” (unacceptable), 4–6 is “acceptable,” and 7–9 is “good work.” We present the Total FIMP Score, which is the mean of the sum of the scores from the five categories on a given session.

Studies have evaluated the predictive validity of FIMP with the hypothesis that high levels of fidelity scored from session observations account for significant variance in pre/post-change in parenting practices, the putative mediator of child outcomes, as well as change in distal child behavior outcomes. The first validity test of FIMP was based on an efficacy trial with an at-risk prevention sample of stepfamilies (Forgatch et al. 2005). Ratings were scored from 40 sessions of 20 families receiving intervention from four practitioners. As expected, high FIMP scores assessed from video recordings of intervention sessions predicted pre/post-improvements in parenting practices assessed from video recordings of parent-child interactions (Forgatch et al. 2005). These findings were replicated in a Norwegian implementation study with 110 therapists and 242 families in a clinical sample, showing that high FIMP scores predicted pre/post-change in observed parenting practices (Forgatch and DeGarmo 2011). A separate Norwegian implementation study assessed FIMP three times during intervention in a clinical sample with 331 parents and 134 therapists. The FIMP scores significantly predicted pre/post-change in child behavior as rated by their parents (Hukkelberg and Ogden 2013). Taken together, these studies support the validity of FIMP as a measure of competent adherence to the model in both efficacy and implementation studies.

#### FIMP Coder Training

FIMP coders are certified PMTO therapists with knowledge of the principles and key components. FIMP training requires approximately 40 h. For reliability, three to five sessions are scored; an intra-class correlation coefficient (ICC) score of 70% or higher is required. The model developers trained the leaders of the Norwegian FIMP team in 2003. The Norwegian FIMP team has trained later generations of Norwegian FIMP coders.

#### FIMP Team

Members of the Norwegian FIMP team were selected within the NIT, based on professional competence, high FIMP scores on their work, and personal skills. For the first 10 years, most FIMP coders were psychologists. Thereafter, the team included experienced therapists, educators, and social workers with comprehensive PMTO experience.

#### Maintenance of Reliability

FIMP coders independently score and discuss videotaped examples in regular meetings during each certification period and at the NIT meetings. FIMP coders are also required to reach criteria in an annual international reliability test administered by the developer. Coders who do not reach criteria are given makeup tests.

#### Certification Assessment

Each trainee submits four videotapes from sessions introducing and troubleshooting *encouragement* and *discipline*. The criterion for achieving certification is a score of 6.0 or higher on the mean Total FIMP Score, which encompasses all five categories for each of the four certification sessions. From G2 onward, the Norwegian FIMP team has had the main responsibility for certification. To prevent bias, FIMP coders do not score certification sessions from therapists in their region. When there is uncertainty about pass or fail certification status, the FIMP team determines the outcome in consensus discussion. In case of disagreement within the team, the two FIMP leaders jointly decide the final scores. If a session tape receives a failing score, the trainee must submit another session tape.

## Results

Table 1 provides outcome data for adoption, sustainability, reach, and fidelity for the seven generations of participants. Data include the year each generation certified, number of therapists who started training, completed with certification, and the percentage certified, number of new participating agencies, number and percentage of therapists still active and practicing with certification and number of years since certification, number of agencies recruited and number still active, therapist educational levels, distributions of therapists in clinical and primary service agencies, number of recruited municipalities and continued activity, and therapists’ FIMP scores at certification.

**Table 1:** Implementation outcomes by generation

Outcomes	Adoption		Sustainability		Reach								Fidelity
Generation (certification year)	Therapists # certified/start (%)	# New agencies	#Active therapists/% (# years)	#Agencies start/active (%)	Educational categories/ Certified therapists		Distribution therapists National level Clinical services		Distribution therapists Municipal community level Primary services			Municipalities (still active)	Mean Total FIMP scores (SD)
					1/%	2/%	#Mental health (%)	#Child welfare (%)	#School counseling (%)	#Child welfare (%)	#Health (%)		
G1 (2001)	34/40 (85)	19	9/24 (17)	19/12 (63)	53	47	24 (70.6)	10 (29.4)	0	0	0	0	6.94* (1.04)
G2 (2003)	79/82 (96.3)	33	29/37 (15)	33/27 (81.8)	17	83	41 (51.9)	38 (48.1)	0	0	0	0	6.34* (0.71)
G3 (2006)	69/69 (100)	45	39/57 (12)	45/32 (71.1)	10	90	5 (7.3)	4 (5.8)	12 (17.4)	35 (50.7)	13 (18.8)	23 (21)	6.95 (0.62)
G4 (2009)	111/121 (91.7)	28	67/61 (9)	28/21 (75)	13	87	27 (24.3)	55 (49.6)	11 (9.9)	10 (9.0)	8 (7.2)	11 (9)	6.89 (0.56)
G5 (2011)	73/82 (89)	30	54/74 (7)	30/21 (70)	9	91	7 (9.6)	21 (28.8)	7 (9.6)	27 (37)	11 (15.0)	17 (12)	6.80 (0.40)
G6 (2015)	90/92 (97.8)	41	81/90 (3)	41/33 (80.5)	13	87	5 (5.6)	9 (10.0)	19 (21.1)	36 (40.0)	21 (23.3)	31 (28)	6.93 (0.43)
G7 (2017)	35/35 (100)	19	35/100 (1)	19/18 (94.7)	18	82	3 (8.6)	2 (5.7)	7 (20.0)	14 (40.0)	9 (25.7)	9 (8)	7.03 (0.41)
Status	491/521 (94.2) Total (%)	215 Total	314/64.0 (9.1) Total (mean)	215/164 (76.3) Total (%)	16 Total	84 Total	112 (22.8) Total	139 (28.3) Total	56 (11.4) Total	122 (24.9) Total	62 (12.6) Total	91 (78) Total (active)	
									Combined primary services 240 (48.9)				

*FIMP scores for G1 and G2 from Forgatch and DeGarmo (2011)

### Adoption Outcomes

Over two decades, 491 of 521 (94.2%) therapists completed the extensive training program with certification. The percentage of completers by generation ranges from a low of 85% for G1 trained by the developer’s team to a high of 100% trained by Norwegian teams. The mean percentage rate of completion when practitioners were trained by the Norwegian team was 95.8%. These data show program adoption to grow with each generation with high numbers of therapists participating and substantial numbers completing the training with certification.

Of those who began training, 30 (5.8%) did not certify. Reasons for not completing (*n* = 19) included change of job, moving, illness, and other personal issues. Trainees with failing certification sessions (*n* = 75) are given three opportunities to resubmit. They receive comprehensive written feedback describing what must be changed and are offered extra coaching. Of the 502 candidates who submitted tapes for certification, only 2.2% (*n* = 11) never achieved a passing score. Number of agencies providing services increased from 19 agencies in G1 to 164 active agencies through G7.

### Sustainability

Of the 491 PMTO therapists, 314 are still certified and practicing, a 64% retention rate. On average, these practitioners have worked with PMTO for 9 years, ranging from 1 to 17 years. Of the 215 recruited agencies, 164 have active PMTO practitioners, a retention rate of 76.3%.

### Reach Outcomes

Reach hypotheses take into account the longer-term implementation goal to shift from a clinical to a more preventive focus. This led to the following: (1) practitioner educational levels will decline and (2) service agency diversity will increase and shift from national to municipality levels. Data support the hypotheses:

1. Educational levels: In G1, 53% of the practitioners had Category 1 backgrounds. For the six subsequent generations, 13.3% had Category 1 educations. The Category 2 percentage increased from 47% in G1 to a mean of 86.7% for G2–G7, fluctuating between 82 and 91%. This reflects the education of primary service practitioners, who tend to hold bachelor’s degrees.
2. From clinical to preventive services at national and municipal levels: In G1 and G2, 100% of the practitioners were from clinical services at the national level. In G3, 86.9% of the trainees were from primary services, with 7.3% from national mental health and 5.8% from child welfare. In G4, ministerial guidelines specified a need for more trainers and coaches, which resulted in an increase in participants from national child welfare (49.6%). From G5–G7, 61.6 to 85.7% were recruited from the primary services in the municipalities. Diversity of agencies from G1 to G7 increased from 24 to 112 PMTO practitioners employed in mental health out-patient clinics, from 10 to 139 in child welfare agencies, and from zero to 240 in primary service agencies. During this period, approximately 22,000 families received PMTO in mental health, child welfare and 78 of 422 municipalities from all regions in Norway.

### Sustained Fidelity

This hypothesis tests the extent to which high intervention fidelity is sustained beyond the three generations previously studied. As Table 1 shows, therapists in each generation achieved scores at or above the required minimum for certification (i.e., 6.0), indicating that fidelity was sustained across all generations. The fidelity scores for G3 in the current study includes 18 additional participants than reported in Forgatch and DeGarmo (2011), as they were certified at a later stage; however, the fidelity scores are replicated. The fidelity scores for G3 through G7 have been close to or above the G1 scores at certification and have held stable. Notice the decrease in the standard deviation over time, a positive trend given the stability in Mean Total FIMP Scores.

## Discussion

The aim of the Norwegian implementation was to adopt the PMTO program and assume executive authority to install the program nationwide, with the ultimate goal of extending its reach to families seeking help within clinical and prevention services throughout the nation. To accomplish this, the implementation had to address multiple challenges. First, PMTO would have to be palatable to Norwegian service systems, practitioners, and families so that they would adopt the program. Sufficient numbers of practitioners would have to be trained to certification with high levels of intervention fidelity. Reach would have to be extended to cover a range of services from clinical to preventive populations throughout the nation. Then, it would be necessary to sustain the quality of training and coaching for multiple generations of therapists. To maintain growth and continued practice with intervention fidelity, an implementation infrastructure would have to be established and maintained. Finally, practitioners would have to be motivated to sustain certification to provide services with intervention fidelity.

Important success factors in achieving these goals included building and maintaining high levels of intervention and implementation competence among each PMTO generation. G1, who would have an essential role in the scale-up, were recruited mainly from mental health and child welfare clinical services. The most qualified were selected to become members of the NIT; others were chosen to establish clusters of trainers and coaches for subsequent generations within their sites. From G3 onward, recruitment was from primary services in the municipal communities, predominantly working at the selected and indicated levels of prevention. From each generation, the NIT has selected the most competent to become trainers and coaches for following generations. This approach to building capacity and increasing the implementation infrastructure has advanced the scale-up.

The NIT has integrated PMTO into key systems serving families: clinical services at the national level within child mental health and child welfare, and a variety of primary services in the municipalities. The stability of the NIT, which consisted of the same persons during the first 10 years, has been an important success factor. Today, it consists of 20 highly competent and committed professionals. The NIT has provided a powerful foundation for the nationwide implementation activities, including the establishment of training and certification protocol for trainers, coaches, and practitioners.

### Sustained Adoption

The Norwegian implementation has produced widespread and long-lasting adoption of PMTO. We assessed uptake in terms of growing numbers of certified practitioners and agencies. From 34 G1 practitioners certified in 2001, the numbers grew to 491 certified by 2018, of these 314 are still active. Certified therapists maintained their certification and continued practicing over years. For example, of those trained in G1 two decades ago, 24% maintained their certification, and practitioners from subsequent generations have kept up their certification with excellent retention rates. An important implementation strategy included recruiting clusters of certified therapists to serve as trainers and coaches in each geographical area to enhance sustainability and promote quality service (e.g., Patras and Klest 2016).

Mandatory participation in video-based coaching from training through maintenance may support this high rate of certified practitioners and ensure that they maintain PMTO practice after training. In turn, this may contribute to continued high levels of fidelity. In addition, it may promote local, regional, and national professional and social commitment and provide practitioners with a sense of belonging to a larger community.

### Extending Reach

The implementation strategy was to build a base of highly competent professionals experienced with clinical samples in the first two generations, some of whom would form the NIT. In turn, they would provide executive leadership to extend the program into agencies offering preventive services. Slightly more than half of G1 had advanced levels of university education and all were drawn from clinical services at the national level. In subsequent generations, although there was a substantial drop in level of education, fidelity levels were sustained and most practitioners were employed by community agencies in the municipalities. Building capacity and leadership in local implementation teams established a foundation for the nationwide scaling up and supported the extension and adaptation of PMTO to prevention services in the municipalities and to some degree to clinical services at the national level.

### Sustained Fidelity—Preventing the Scale-Up Penalty

High levels of fidelity were sustained from one generation to the next across seven generations, which may have prevented a common problem. Following implementation, there is a well-known scale-up penalty in which effect sizes shrink (Institute of Medicine and National Research Council 2014). Weisz et al. (2014) refer to this as “the implementation cliff.” When ESIs are transferred from the ivory tower to the complex and dynamic contexts of community service agencies, they tend to suffer a slow death as practitioners make adaptations to the original model, and the positive effects shrink during the scaling-up process (Curtis et al. 2004; Fixsen et al. 2005; Weisz et al. 2015). A drift from model fidelity may explain the decay in effect sizes (Dodge 2001; Welsh et al. 2010). Such a decline in effect size was not found in a study evaluating Norway’s nationwide implementation, however. Tømmeraas and Ogden (2017) examined effect sizes of family outcomes based on three generations of therapists offering PMTO in diverse community service systems throughout Norway. Effect sizes for child outcomes were sustained for more than 4 years in spite of increased heterogeneity of client characteristics, level of practitioners’ prior educational training, and type of delivery system.

Sustained fidelity may have been affected by a variety of factors. For example, when fidelity ratings are based on direct observation of therapy sessions, therapists must show that they are practicing the model as specified. The requirement to participate in coaching sessions following certification based on observation may also sustain high levels of fidelity. The use of a fidelity measure with strong predictive validity is still somewhat rare in implementation studies.

### Challenges and Lessons Learned

This nationwide implementation of the PMTO model taught us many lessons. Challenges presented by some from the G1 certified practitioners (e.g., refusing to follow the quality assurance criteria, not accepting the coach they were assigned) led the NIT to clarify qualifications and personal characteristics that are required in the recruitment of new generations. Over the years, NIT has specified several selection characteristics and become more active in the recruitment process.

Obtaining and maintaining commitment and support from agency leaders continues as a challenge. It has helped to require that each service agency provide written agreements that enable their candidates to fulfill the demands of the training program and participate in the long-term implementation process. For instance, the top administrative directors and executive leaders agree to permit the trainees to spend a minimum of 40% of their working time participating in training activities and offering PMTO services to at least five families per candidate.

Mandatory participation in coaching groups presented a challenge. During the early generations, agency leaders claimed that coaching was too time consuming, especially for trainees who had to travel far. NCCBD addressed this problem by recruiting more candidates from the same geographic area and providing local coaching groups. Regular local coaching for trainees throughout their 18-month training period has promoted high rates of certified therapists and high levels of fidelity. Practitioners are supported by local coaches and trainers, who are supported by the NIT, a process that contributes to the maintenance of certified therapists. Strong local networks, as well as regular regional and national boosters, provide local practitioners with a sense of belonging to a larger community. All share the mission of offering effective and evidence-based intervention for children and families.

Maintaining motivation at every level in the PMTO system is a challenge that is addressed by the NIT’s attention to nationwide connectivity. Practitioners are also inspired by findings from the studies conducted by the NCCBD research team with positive outcomes for children and families and successful adaptations of the program (e.g., Ogden and Hagen 2008). Furthermore, positive family feedback to their therapists maintains practitioner enthusiasm.

### Limitations

One limitation in this study is that fidelity was assessed only at certification. Regular fidelity assessment should take place to ensure sustained intervention fidelity (Sigmarsdóttir et al. 2018). Without research funding, it is resource demanding to assess therapists’ fidelity after certification. FIMP validation studies, however, have shown that changes in pre/post-parenting practices and child outcomes were predicted by clinicians during their regular practice, not just at certification (Forgatch and DeGarmo 2011; Hukkelberg and Ogden 2013). More studies should test if there is a decline in fidelity and drift from the model as therapists continue their practice.

## Conclusion

The Norwegian program marked the first time the developers of GenerationPMTO implemented the intervention beyond their own team. Although they had conducted multiple efficacy trials at clinical and prevention levels (see Dishion et al. 2016), they had little experience with real-world settings. The lurch into a nationwide program was not only ambitious, it also generated the full-transfer approach, a method that grew out of the collaboration between the program developer and the Norwegian team. The approach has since been applied by GenerationPMTO teams in other implementation countries and sites (see Sigmarsdóttir et al. 2018). Data from the present study indicate that the full-transfer approach to implementation yields promise as a means of scaling-up with sustained fidelity to an increasing number and diversity of therapists and service levels.

The Norwegian program has benefitted from strong and reliable funding provided to a coordinating center, NCCBD. In turn, NCCBD has served as the home for implementation and research activities that evaluate processes and outcomes, as well as provided for further development, adaptation, and quality assurance. Some may claim that the certainty of stable financial and political guarantee in the form of a fixed item on the national budget has ensured the success of the implementation. Indeed, this has facilitated the quality of work and the capacity to conduct extensive research. However, a favorable financial situation is not enough to achieve goals that enhance adoption, such as training programs that result in large numbers of practitioners who achieve certification and continue their practice in the model with high fidelity. Without such a growing force of committed and competent practitioners, extending the program’s nationwide reach across multiple service sources would be unlikely.

The sustainability of PMTO in Norway over nearly two decades rests on a strong infrastructure, carefully articulated implementation strategy, collaborative relationship between the developer and the adopter, continuous economic support from governmental grants, and careful attention to intervention fidelity for each new generation of therapists. Over the years, the core principles and procedures of the PMTO program have been maintained without substantial changes. Norway has installed and maintained effective PMTO in environments dedicated to adopt and nurture the model and the practitioners.
